# High Monocyte Count Associated with Human Cytomegalovirus Replication In Vivo and Glucocorticoid Therapy May Be a Hallmark of Disease

**DOI:** 10.3390/ijms23179595

**Published:** 2022-08-24

**Authors:** Przemyslaw Zdziarski, Andrzej Gamian

**Affiliations:** 1 Lower Silesian Oncology, Pulmonology and Hematology Center, P.O. Box 1818, 50-385 Wroclaw, Poland; 2 Hirszfeld Institute of Immunology and Experimental Therapy, Polish Academy of Sciences, 53-114 Wroclaw, Poland

**Keywords:** virome, cytomegalovirus (CMV) kinetics, definitions of cytomegalovirus disease, virus replication, clinical model/translational medicine, homogeneous sample of subjects logarithmic phase of infection, innate immune response, monocytes characteristics and analysis, CD14, monocytosis, mononucleosis

## Abstract

Cytomegalovirus (CMV) syndrome and infectious disease are defined as pathogen detection with appropriate clinical symptoms, but there are not pathognomonic signs of CMV disease. Although the prodrome of acute minor viral infections leukopenia (lymphopenia and neutropenia) is noted with onset of fever, followed by monocytosis, the role of monocytosis in CMV disease has not been described. Furthermore, under influence of corticosteroid therapy, CMV reactivation and monocytosis are described, but without a strict relationship with steroids dose. In the study, the monocyte level was investigated during the CMV infectious process. Regrettably, a non-selected group of 160 patients with high CMV viremia showed high dispersion of monocyte level and comparable with the median value for healthy subjects. Therefore, we investigated monocyte level in CMV-infected patients in relation to the logarithmic phase of the infectious process. Samples from patients with active CMV replication (exponential growth of CMV viremia) were tested. Significant monocytosis (above 1200/µL) during the logarithmic phase of CMV infection (with exponent between 3.23 and 5.77) was observed. Increased count and percentage of monocytes correlated with viral replication in several clinical situations except when there was a rapid recovery without relapse. Furthermore, glucocorticoids equivalent to 10 and 20 mg of dexamethasone during a 2–3-week period caused monocytosis—significant increase (to 1604 and 2214/µL, respectively). Conclusion: In light of the logarithmic increase of viral load, high monocytosis is a hallmark of CMV replication. In the COVID-19 era, presence of high virus level, especially part of virome (CMV) in the molecular technique, is not sufficient for the definition of either proven or probable CMV replication at any site. These preliminary observations merit additional studies to establish whether this clinical response is mediated by monocyte production or by decrease of differentiation to macrophages.

## 1. Introduction

Host–microbe interactions shape the phenotype, activity, reciprocal adaptation to adverse environmental conditions, differentiate, and ontogeny and phylogeny metabolisms, giving rise to the theory of coevolution of microbiota and their host. The virome is the new field of research. It has already delivered novel and important concepts for describing host–microbial interactions, but with rapid growth in interest covering different fields. Therefore, the redefinition of basic terms such as “microbiome”, “microbiota”, “infection”, “virome”, “replication”, “infectious disease”, “mononucleosis/monocytosis”, and “opportunistic infection” is very important [1,2].

The human herpesvirus, especially cytomegalovirus (CMV), is the most interesting part of human virome and CMV may be a model virus in virome research, because CMV is a very immunoreactive virus. The range of CMV infection is wide, from CMV reactivation, presenting mainly as asymptomatic viremia to CMV end-organ diseases, such as esophagitis, gastroenteritis, hepatitis, retinitis, pneumonia, and encephalitis, but without strict laboratory criteria [2]. 

Although terminology of the infectious CMV process was prepared for clinical trials in transplant recipients, “CMV disease” and “CMV replication” are less clearly defined: “CMV replication” was described as evidence of viral multiplication without accurate measure, therefore sometimes being misused instead of “CMV infection” as described for transplant patients [2]. Interestingly, the trend line of cumulative incidence of CMV reactivation and CMV disease show near the same shape, the reactivation was observed earlier [3].

Although CMV viremia continues to be the most common cause of medical intervention after transplantation, a definite cut-off for CMV DNA load and strict influence of steroids therapy has not been established at the present time [2,4]. While studies for the explanation of CMV infectious process were conducted, the clinical situation is still difficult in the interpretation [5,6]. 

Firstly, it is impossible to exclude all other causes of the clinical symptomatology described as CMV syndrome, patients receive several immunomodulatory drugs (e.g., steroids) and intra-host CMV diversity may be the source of atypical clinical manifestation [7,8]. Secondly, in vitro reactivation of the virus is induced in experimentally infected granulocyte-macrophage progenitors by co-cultivation with permissive cells or by treatment with proinflammatory cytokines [9]. Thirdly, the single positive result of polymerase chain reaction (PCR) may be difficult to interpret as a virome or not with latent viruses such as CMV. All patients previously infected will have a virus present in blood or tissue, irrespective of whether they have a disease or not [2,3,5]. It is generally found that patients with active CMV have a much higher viral load, but an appropriate cut-off level has not been universally described [2,10]. It is crucial that the detection of virus, antigen, or DNA with very sensitive methods in blood does not mean that CMV is currently in a replicating phase [8]. Therefore, there is a significant gap of knowledge. 

In contrast to the rapid development of molecular background, basic understanding of physiology, symptomatology, and differential diagnosis of CMV disease is still an open question [2,3,6,7,10,11]. Unfortunately, sometimes CMV reactivation is arbitrarily defined as CMV viremia >1000 copies/mL [12] in spite of the fact that viral particles may be in free form or within leukocytes and deep leukopenia may affect the absolute result [13]. Such studies show ambiguous results with nearly the same prevalence of no CMV infection and CMV reactivation in recipient positive donor-negative constellation (R+/D−), i.e., 4.2% and 4.3%, respectively [12]. Furthermore, CMV disease defined in this way (CMV > 1000 copies/mL and pneumonitis or gastrointestinal disease) was observed only in the case of the D+R+ constellation. No cytomegalovirus disease was observed in D−R− or D−R+ pairs, contrary to the accepted rule that in patients undergoing allo-HSCT, the use of a CMV (−) donor to a CMV (+) patient (D−/R+) has been associated with an increased risk for non-relapse mortality and decreased survival [2,3,13]. The risk of reoccurrence the D−/R− is less than D+/R− (3.1 vs. 12.9%) as presented in Styczyński et al. [3]. Other infectious causes of pneumonitis or colitis in such immunodeficient patients were not analyzed and no patients with CMV disease developed relapse as described by Park et al. [12]. For the development of CMV disease (as a virome dysbiosis), not only the size of the viral load is crucial, but also the parameters of the host, such as IgG, leukocyte level, especially lymphopenia [1,14,15]. 

Although the adaptive immune response to CMV is a well described phenomenon, in a clinical situation, innate immune response precedes antigen presentation, lymphocyte recruitment, and effector immune response [14]. Furthermore, although CMV mononucleosis diagnosed by reactive lymphocytes under the influence of antigen-presenting cells, little attention has been paid to the function of monocytes [16,17]. Short-lived monocytes circulate in the bloodstream and only infiltrate into secondary lymphoid organs and diverse tissues, but due to biopotency and immunological function, monocytes are targets of CMV to thereby evade and manipulate immune responses, as reviewed elsewhere [17]. The increasing monocyte count may be (apart from increased monocytopoiesis) the result of half-life greater than 3 days and mobilization of peripheral pool or reduced recruitment into tissue macrophages. Latent infection of granulocyte-macrophage progenitors is crucial for the infectious process and subsequent replication [17,18]. It corresponds with data showing that grafts from CMV-seropositive donors (D+) contain more antiviral cytokines: chemokine macrophage inflammatory protein 1β and TNF associated with the antiviral process, produced mainly by active monocytes and macrophages [19]. In this light, modification of monocytosis may be an important component of the pathogenetic chain but has not been studied so far. 

Although in clinical observations, monocytosis is well described under influence of corticosteroid therapy and corticoids are the risk factor for CMV reactivation, until now, high monocytosis as an intermediary pathogenetic link has not been considered. Notably, in medicine (especially clinical immunology and transplant practice), the relationship between monocyte level and steroids dose are not studied [20]. 

The aim of our retrospective analysis was to check whether CMV infectious process, especially virus replication, causes changes in the number of monocytes and whether excessive glucocorticotherapy may be a factor modifying monocytosis and, in this manner, CMV disease. 

## 2. Results

**Figure 1:**
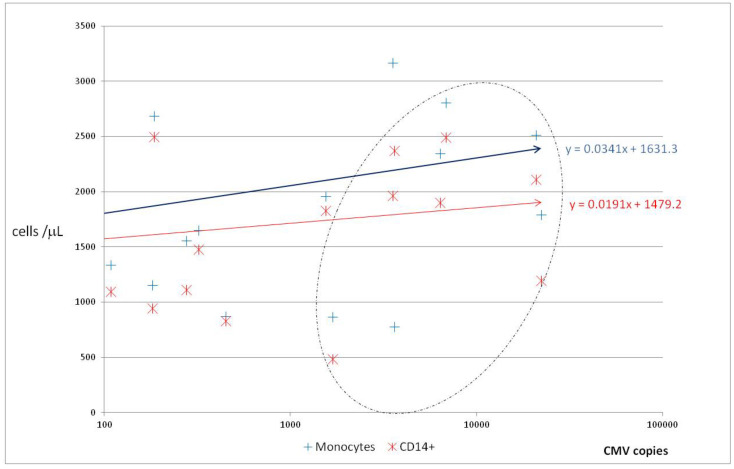
Regression and relationship between monocyte level (assessed with two methods) and CMV viremia—presented on vertical axis in logarithmic scale. Monocyte (hematology autoanalyzer), CD14+ cells (flow cytometry) counts are expressed on *y* axis.

In the preliminary study, the sequential analysis of CMV-viral load in all participants (see Material and Methods) showed poor or low correlation with clinical manifestation, total monocyte count, and WBC subsets. The correlation coefficients between viremia and monocytosis defined (1) morphologically and/or (2) by flow cytometry (CD14+ level) cells were r = 0.3233, 0.2140, respectively. Interestingly, under the influence of viremia growth, the increase was observed for primarily in total monocytosis. Although CMV-infected monocytes are required for viral replication [17], patients presented various degree leucopenia and CMV copy number was usually higher than WBC, especially monocyte count (Figure 1).

The regression line of absolute monocyte as well as CD14+ levels showed increasing tendency with nearly the same gradient, whereas correlations with viremia were poor (correlation coefficient r = 0.3233 and r = 0.2140, respectively). In contrast to total monocyte count, the viremia level did not form continuous data. In the range of 500–1500 CMV copies, a clear gap was observed. This indicates that, in contrast to the absolute value of CMV copy number, the exponential increase of viral load in timeline is a good measure of replication. The area marked with a dotted line corresponded with higher CMV copy number than monocyte count. 

Free, extracellular viral particles do not replicate. This prompted the use with ≥100 CMV copy number/10^5^ per nucleated blood cells (NBC) as described previously in our center [15]. In such situation, the patients were subsequently treated with a preemptive regimen of ganciclovir or valganciclovir because they were designated as having clinically significant CMV risk [2,3,16,17]. Therefore, the patients were disqualified from further analysis. A minority of patients (16/160) had low viral load values that did not require preemptive therapy, followed by exponential increases of viremia in the subsequent analysis (see Materials and methods). This exponential increase in viral load was therefore used as a criterion in selecting patients who undoubtedly had virus multiplication (replication), probably in blood. When in such patients the exponential function of viremia was analyzed with base = 10 (common logarithm), the exponent x was between 3.23 and 5.77 (1). With this technique and the patient selection algorithm, it proves that there is more than 1 virion in one cell due to active CMV replication (multiplication machinery) (Figure 1).

### 2.1. Monocyte Analysis by Various Techniques during Exponential Viremia Growth—Significant Monocytosis as a Hallmark CMV Replication

In preliminary research, a simple gating step for forward and side scatter (FSC/SSC) for monocyte analysis was not useful in our patients (data not shown). Therefore, a cytometric method based on CD45/SSC was used [15,21,22,23]. It showed a very high correlation with the results obtained with the hematology analyzer. Both techniques were slightly less correlated with manual counting. The correlation coefficient between the two cytometry-based techniques was r^2^ = 0.97, but interestingly, the highest correlation was observed between hematology analyzer and CD45/SSC gating (Table 1). 

The Pearson r^2^ coefficients are presented. Two techniques were based on morphology of monocytes in contrast to two cytometric methods (CD14-based or CD45/SSC).

(a) manual microscopic analysis of slides from May–Grünwald–Giemsa stain (MGG smears)
(b) hematology analyzer

**Figure 2:**
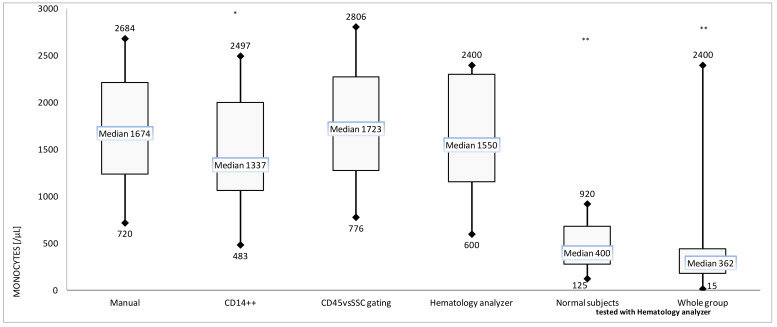
Box and whisker plot for the monocyte level in patients with CMV infection in exponential phase of infectious process; * *p* < 0.05; ** *p* < 0.01.

Our analysis also showed good correlation and concordance between cell count based on WBC enumeration and two cytometric approaches to monocyte definition (1) CD45 vs. SSc gating (2) CD14++ mononuclear cells. Manual counting deviated from the other techniques (Table 1). No significant difference was observed between manual, hematology analyzer, and CD45/SSC-based method, but unfortunately CD14-based flow cytometric method underestimated the monocyte level (Figure 2).

For comparison, the monocyte level in CMV-sero-positive normal subjects without active replication (latent infection) was presented. Notably, when we look at monocyte level in a total group of 160 patients with viremia (in different phase of CMV disease), the median monocyte level was comparable with normal CMV-positive subjects without CMV disease. The data of normal subjects and of the whole group were obtained with a hematology analyzer.

The correlation between the four methods of monocyte counting and viremia level in exponential phase was strong (r^2^ = 0.61214957, 0.572744268, 0.680241725, and 0.662373669 for manual, CD14-, or CD45-based and hematology analyzer, respectively), in contrast to stationary phase of infectious process (Figure 1).

**Figure 3:**
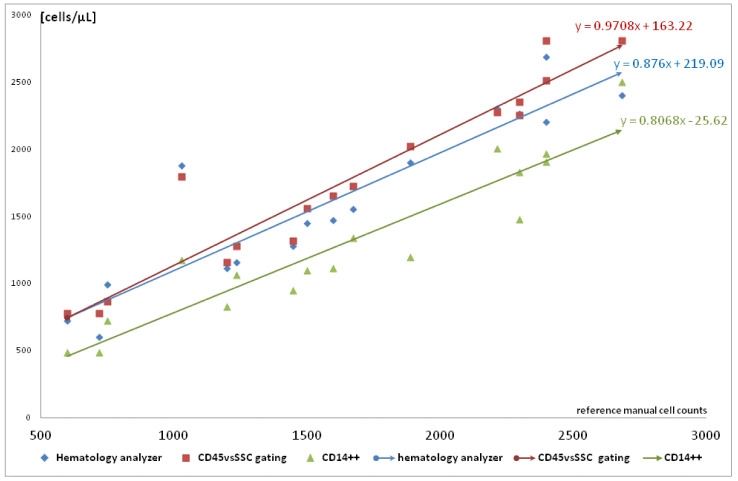
Regression and relationship between monocyte count obtained in manual reference method (*x* axis) and two flow methods: (1) hematology analyzers, (2) cytometry with CD45vsSSC gating, or (3) CD14++ cells presented on *y* axis.

In contrast to the simple correlation presented in Table 1, when we look at the relationship between monocyte count and linear regression, lower values were observed when monocytes were defined as CD14++ mononuclear cells. The scatterplot of the changes in monocyte count is presented in Figure 3.

High accuracy was observed, but Pearson coefficient was higher between two flow methods than between manual and autoanalyzer or cytometry methods (Table 1). The discrepancy was observed especially in patients with high monocytosis. However, CD14+ mononuclear cells showed good correlation with manual counting but absolute values of CD14+ monocytes were approximately 250 cells/µL lower than in the hematology determinations (the trend lines were nearly parallel). Even greater difference was observed between CD14+ monocytes and the gated CD45+/SSC or by manual method (Figure 2).

However, regardless of the technique (morphological or cytometric techniques of monocyte counting), our analysis showed significant monocytosis during active CMV infectious process with exponential growth of CMV-viremia. To compare, the median monocyte levels of sero-positive normal subjects and CMV-positive patients without here-defined active CMV replication were insignificant and about 1000/μL lower (Figure 2). Non-selected whole group of 160 patients with positive CMV results [15] showed high dispersion of monocyte level and comparable with the median value of healthy subjects (Figure 2).

**Figure 4:**
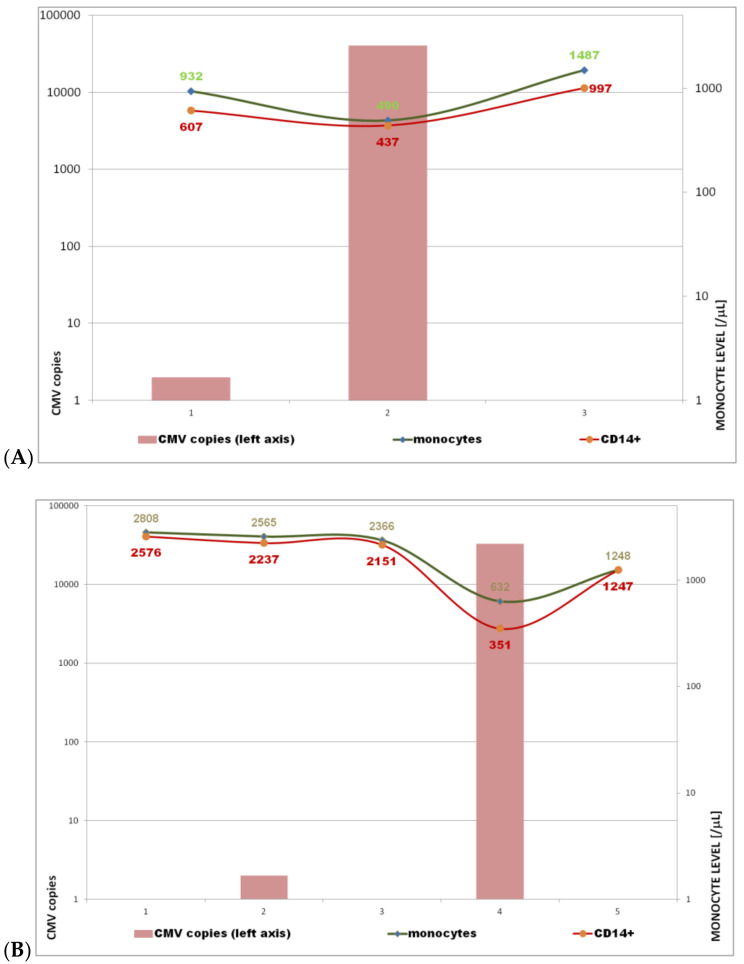

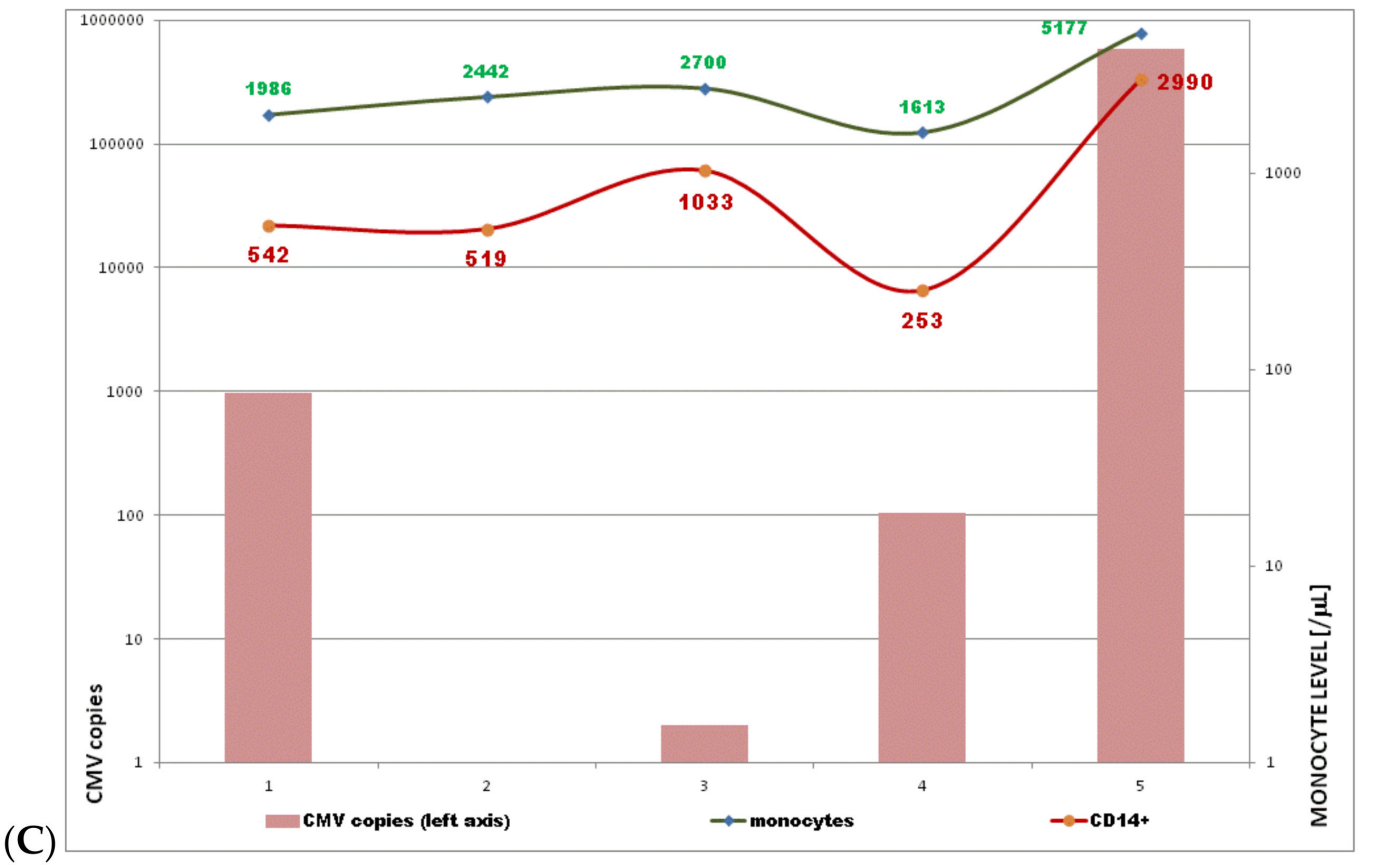
Relationship between CMV-DNA and monocyte count. Monocyte level and CMV copy numbers on 10^5^ nucleated cells of the whole blood (as the box) were expressed on the right and left axis, respectively.] According to the guidelines and diagnostic chain (see section Material and Methods), the CMV monitoring was carried out once a week. The course of CMV disease is presented in the timeline (weekly results are shown on the *x*-axis) until viremia withdrawal or fatal outcome. Exponential function of viremia with various bases (b) that are a positive real number higher than 1 and whole copy CMV number as a values of function f(x) = bx, when b > 1; x > 0. Representative patient plots of each group are shown in 3 clinical situations and outcomes: (**A**) symptomatic infection with rapid latency, exponent x = 4.77. (**B**) oligosymptomatic infection with rapid relapse with intensive symptoms of CMV mononucleosis and good outcome x = 4.52. (**C**) symptomatic primary infection with temporary viremia disappearance, prolonged intensive mononucleosis and relapse with fatal outcome. The exponential growth of viremia was the highest x = 5.77.

Monocytes accounted for up to 25% of leukocytes (WBC) during active replication (data not shown). Generally, large dispersion of monocytosis values was observed (especially in the low viral load) and significant increase of viral load corresponded with low gradient of increase of monocytes count (Figure 1). Therefore, we analyzed the monocyte and viremia fluctuation in several patients with various CMV disease courses and outcomes (Figure 4). Notably, initial low CMV viremia level (<1000/μL) was not tantamount to latency and in contrast to comparable initial CMV load, higher monocytosis was observed in patient Figure 4B than Figure 4A.

### 2.2. Potential Role of Steroids

It is noteworthy that relative monocytosis is also described in minor viral infections (during the prodrome) and chronic corticosteroid therapy, but without respect to the dose of steroids and monocyte count [20]. However, in vitro treatment of isolated monocytes with high dose of hydrocortisone (equivalent to 18.1233 mg/L of dexamethasone) over the period of 6–9 days induced differentiation into macrophages as well as CMV gene expression [16]. 

**Figure 5:**
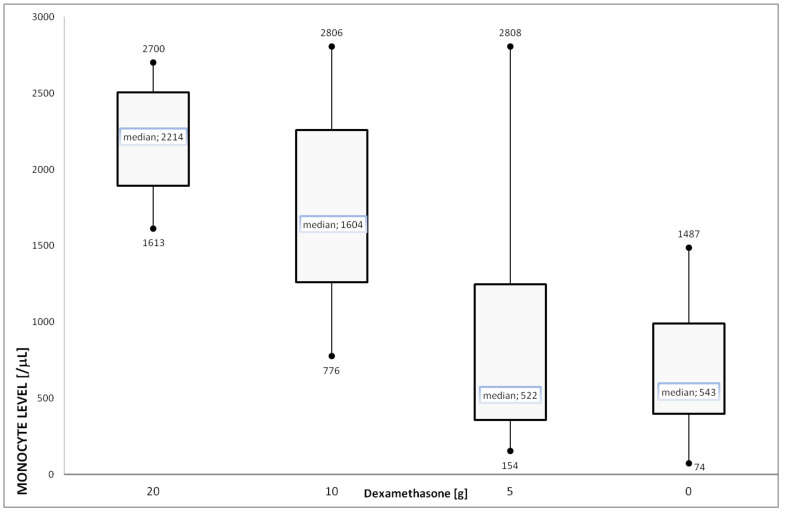
Various monocyte levels (hematology autoanalyzer) under the influence of ongoing glucocorticoid therapy. The dose of steroids was presented as an equivalent dose of dexamethasone that forms a geometric sequence (ar^n^) with common ratio r = 2 and start term a = 5 (exponential growth). For comparison, the subgroups within included patients (see Material and Methods), monocytosis was presented in included patients (also anti-CMV+ and immunodeficient) that had never been treated with steroids (right bar). The interquartile range (IQR) was about 1.5-fold higher for 5 and 10 mg of dexamethasone than 0 and 20 mg.

Patients receiving high dose of steroids developed a higher degree of monocytosis than patients who received no or low dose (Figure 5). Notably, the observed changes affected the absolute value, therefore the amount of granulocyte and lymphopenia did not result in an increase in monocytosis.

## 3. Discussion

Relative monocytosis is usually observed in a chronic disease such as hematologic malignancy, protozoan infection, tuberculosis, or sarcoidosis. In contrast to relative, the absolute monocytosis is therefore a rarely observed phenomenon in the human clinic [20,24]. The absolute number of monocytes and changes in several fractions are a derivative of their marrow production and fast (within a few days) migration into tissues, tightly associated with CMV replication as reviewed by Min et al. [7]. Laboratory norms for monocytosis are evolving with a significant spread between values above 500 or 1200/µL (Wallach’s Interpretation of Diagnostic Tests Ninth and Tenth editions, respectively) [20]. Contrary to lymphocyte subset, the sequential analysis of monocyte counts is not analyzed in CMV disease. Furthermore, because of the use of different antibodies for monocyte identification, the nomenclature of monocytes in human blood has become quite confusing due to the existence of several subpopulations and CD14 use as an exclusive monocyte indicator [24,25]. Although it is the most stable marker of monocyte lineage, it can be downregulated completely by interleukins [26]. This prompts further use of both morphological and cytometric techniques of monocyte counting.

In the present study, monocytes were monitored in a time-lapse manner in patients who developed CMV replication by sequential counting, testing with various classical techniques. Due to divergent conclusions of previous publications and poor correlations between monocyte and viremia level (Figure 1), a new attempt was made to standardize the examined patients. Active phase virus replication was demonstrated by exponential function of viremia growth (e.g., Figure 4a–c with exponent 4.52–5.77).

### 3.1. Monocyte Quantitation, Description with Different Methods: Slight Influence of the Basic Characteristics of Cells on CMV-Induced Monocytosis Finding

After hematopoiesis from myelomonocytic stem cells in bone marrow, monocytes move into the blood where they circulate with a half-life of 1–3 days [17]. Monocytes are the cells known to hematologists for a century as monocytes on the basis of the structure. The result of a morphology-based examination is a leading parameter in clinical situation, especially in critically ill patients, when more specific enumeration and analysis by flow cytometry is too long for medical decision and preemptive therapy. In addition, most of the known causes of monocytosis in the clinic relate to conditions defined many years ago based on morphology (morphometric criteria of WBC) rather than the expression of surface markers [24,25,26].

Automatic analysis in the case of severe leucopenia (e.g., WBC < 100 cells, monocyte count < 10) is problematic, the analysis requires examination of a larger blood volume or manual analysis MGG smear. In our observation, the entire dataset from comparison presented in Figure 2 and Figure 3, monocyte count analyzed simultaneously on hematology analyzer, flow cytometry (with CD45-gating or CD14+), and manual differential counts showed a high correlation coefficient (Table 1). Although CD14 expression is similar in fresh blood and Ficoll-isolated monocytes, the enumeration with cytometric methods using different standard beads is laborious and time-consuming [27]. In our observation, monocytosis was observed regardless of the used technique (Figure 2) and may be the hallmark of active replication. Several methods could be used for quantitation of monocytes but cell counting is a somewhat non-repeatable model [22,28]. Therefore, the standard approach in clinical manual analysis and hematology analyzers relies on physical properties of these cells including light scatter. Our preliminary analysis with forward and side scatter (FSC/SSC) examination of monocytes in our patients was not useful because it did not discriminate well between leukemic blasts, lymphocytes, and monocytes [29]. When living cells are gated depending on SSC, lymphocytes and monocytes do not form readily distinguishable populations [25,30]. Furthermore, the large proportion of the studied patients (Table 2) as well as significant part of patients with cytomegalovirus disease are hematological patients, i.e., with lymphoproliferative disease (lymphomas) and with adaptive immune response disorders (primary or secondary immunodeficiency). These laboratory and terminology (monocyte definition) difficulties were the main reason for using various techniques, both morphological (available in every hospital) and cytometric (existing in hematology centers but not always for AIDS) [31]. Our observation of good accuracy of CD45/SSC gating and other methods (Figure 1 and Figure 2) corresponds with other studies on hematologic diseases. As described by Lacombe and coworkers, they discriminate well between monocyte and leukemic blasts, that are CD45 low [22]. Although correlation between flow hematology analyzers and manual MGG smears is high, in a scientific and experimental situation, flow cytometry has been proposed as the reference method for the detection of various cells in blood, for instance monocytes and dendritic cells [27,28,32].

Our observation corresponds with Grimaldi’s report, where hematology analyzer sometimes showed slightly higher monocyte counts than the manual (MGG-based) or CD14-based cytometric method [33]. The new phenomenon was presented here, when hematology analyzer or CD45/SSC gating resulted in higher number of monocytes than simple CD14-based gating, but—notably—in patients with exponential phase only (Figure 2). Further investigation with a larger sample size is warranted. 

Monocytes represent a much more heterogeneous population, and the use of only one cytometric marker without additional gating leads to inaccurate conclusions [29]. We observed higher correlation and efficiency than in comparative study of several hematology analyzers [34]. Interestingly, in our observation regression lines were nearly parallel and their slope coefficients were 0.8068 and 0.876 (Figure 3). One explanation is that monocytes are a CMV Trojan horse [17,35,36,37] as presented in Figure 1 (CMV copy number >> monocyte count). In contrast to comparable initial CMV load, higher initial monocytosis was observed in patient Figure 4B than Figure 4A and clinical sequel as well as viremia level peak were quite different (Figure 4). Monocytes together with CD34+ progenitor cells were found in naturally infected cells [17,18,35,37], but our observation in exponential growth of viremia often corresponded with condition when CMV copy number was higher than monocyte count (Figure 1). 

### 3.2. Clinical Manifestation: Significant Monocytosis under the Influence of CMV Replication

Bone marrow is the primary target of CMV infection, and myelomonocytic stem cell precursors produce a constant number of monocytes from which the stable fraction is infected [17,37,38]. However, lymphocytosis with atypical lymphocytes is a well-known symptom in immunocompetent patients, but most patients with active CMV replication are lymphopenic under the influence of NHL, HSCT, or primary immunodeficiency (Table 2) [6,8]. High monocyte percentage (up to 25% of WBC in our results) corresponds with such situation. The cause of monocytosis is primarily hematologic or infectious disease, but cytomegalovirus disease has not been described [20]. However, when we look at the evolution of absolute monocytosis in specific clinical situations, presented in Figure 4, there is a slight decrease in monocytes in the first phase, followed by a strong increase. The strong correlation between the number of monocytes and the exponential increase of viremia level indicates that high monocytosis (ranging between 1337 and 1723, depending on the method) may be a sign (hallmark) of cytomegalovirus disease under influence of CMV replication (Figure 2). Notably, there are not pathognomonic signs of CMV and Owl’s eye appearance of inclusion bodies is not strictly described in the light of virus cycle (sometimes observed in Reed–Steenberg cells) so CMV disease was difficult for diagnosis [2,3], as well as CMV mononucleosis (i.e., atypical lymphocytes, classified as monocytes in hematology analyzers). Notably, herpesviral mononucleosis (EBV or CMV-induced) is often understood as monocytosis in spite of the fact that the atypical large cells are not monocytes as indicated by automated hematology analyzer (Baker 900 plus or MaxM, Coulter), but reactive lymphocytes [38,39]. Therefore, the clinical description and sign of CMV disease is still an open issue [2,3,6,10]. The median value of monocyte level in the whole group was comparable with healthy subjects with latent form of the infectious process i.e., 362 and 400, respectively (Figure 2). It also indicates that positive CMV results are not synonymous with cytomegalovirus disease and median viremia about 1000/mL may be observed without existing progress of CMV replication (Table 2, Figure 2) so without significant monocytosis, as seen in the regression analysis and the large dispersion of the results (Figure 1). In addition, low copy value, i.e., lacking CMV copies in small blood sample (or having <100 CMV copies/10^5^ NBC or viremia level <1000/mL), does not mean latency (no virus replication as presented in Figure 4) so virome homeostasis.

Conversion of numerical data into their logarithms is sometimes used in the case of strongly asymmetric distributions and in the case of large differences in the size of the analyzed numbers, as well as in situations where the aim of the study is to determine the ratio of numbers, not their difference. Such situation is observed in infectious process (i.e., bacterial growth as well as virus replication), for the first time presented here for CMV—absolute numbers form an exponential, not an arithmetic sequence and non-continuous data (Figure 1, graphical abstract). The same rule and significant monocytosis are not observed when we look at viremia in the stationary phase in the same patients and all patients (whole group) with positive CMV results (Figure 2) [10,11]. The reason may be the changes in the number and distribution of leukocytes due to the applied treatment for example myelotoxic drugs, such as ganciclovir (Table 2). It is worth noting that secondary immunodeficiency patients in the valgancyclovir arm experienced a statistically significant early and prolonged decrease in their monocyte counts followed by a transient increase during the post-treatment and an increase in absolute neutrophil counts [40].

The relationship of monocytosis and CMV-viremia in stationary phase (Figure 2) or in single measurements (Figure 1) was negligible. Single results of viremia (even >1000 copies) also did not reflect interactions between CMV and monocytes and correlations were poor (r = 0.3233 or 0.2140, Figure 1). Kinetics (hence an exponential relationship) created a more reliable image of the new, extremely dynamic interaction (e.g., presented in Figure 4). Therefore, preemptive CMV monitoring with time-lapse examination of viremia level (once weekly for 12–16 weeks for SOT [41] or later for HSCT [14,15]) is the new approach. Together with the monocytosis examinations presented here, it creates an opportunity for monitored therapy and further observations.

The significant increase and exponent of the viremia function may be a new indicator of CMV disease, most likely incubation period. 

### 3.3. Potential Role of Steroids

The third explanation of monocytes increase with CMV may be in part the influence of long-term immunosuppressive steroids. Therefore, we compared monocytes level in 8 patients (selected from the first group of 16 patients) in latent phase with different doses of steroids converted to dexamethasone (Table 2) [42]. The effects of steroids and CMV replication were therefore observed separately (see Material and Methods). Although patients received high dose glucocorticoids in various periods and for various indications, the virus replication (defined as an exponential viremia growth) was not observed, but low median monocyte level was observed, contrary to active replication period (Table 2, Figure 5 right bar). Then, we compared the geometric progression of dexamethasone dose with the values of monocytes per 1 μL: it formed also geometric sequence with common ratio about 2. The rule was observed at the highest doses (i.e., 10–20 mg). In extremal value of monocytosis, 5 mg of dexamethasone gave the two-fold adjusted monocyte count (higher or lesser for maximum or minimum, respectively) than observed in the control group of CMV-positive patients without corticotherapy, i.e., 154 and 2808 vs. 74 and 1487, respectively (Figure 5). When we look at median value, steroid equivalent to 5 mg of dexamethasone did not cause any significant effect in immunodeficient patients (Figure 5) and median value of monocytosis was comparable with anti-CMV+ healthy control (522 and 400 monocytes per µL—Figure 5 and Figure 2, respectively). 

To our knowledge, this is the first clinical presentation of dose-dependent effect of long-term steroids on monocyte level in patients with latent CMV. It is not to be underestimated in the therapeutic application of dexamethasone in hemato-oncological chemotherapy. The synthetic glucocorticoid dexamethasone is a commonly administered antiemetic, but in short-term therapy. In contrast, there was a dose-dependent reduction in counts of monocytes, 4 h after dexamethasone, followed by a rebound increase in cell counts at 24 h, but later cell counts were similar to baseline levels [43].

### 3.4. Virome Research in Translational Medicine

The division of microorganisms into beneficial, pathogenic, and neutral according to microbial interactions with their hosts is very difficult for virome [1]. Although the CMV entry is non-symptomatic, the latent form CMV modifies many host genes expression and pluripotent hematopoietic stem cells as described previously [16,17,35,44]. The primary infections occur during pregnancy or infancy and CMV incidence decreases with age [14,29,45]. CMV is a key element for the development of immunity: physiologically up to 10% of CD8 cells are CMV specific. This percentage will increase in CVID patients (IgG-deficient) with narrow lymphocyte repertoire (10.8%) [46]. The beneficial CMV role is finished in the state of significant primary or secondary immunodeficiency. When we look at clinical sequel and the fact that low viral load does not mean latency (Figure 4), the virus replication may be crucial for outcome of immunodeficiency. The CMV fatal outcome corresponds with severe immunodeficiency (usually CD4 < 100 cells/μL) and virome, so CMV in particular seems to be an evolutionary mechanism for eliminating immunocompromised individuals. The greater the deficit, the faster the selection (Figure 4). The same may be observed in the general population: the coexistence CMV reactivation with AIDS (grade 3) and prevalence in developing countries with higher microbial pressure. On a cellular level, such selection may be observed in the monocyte population: significant decrease and lowest monocyte level correspond with fatal outcome later (Figure 4C). Subsequent monocyte growth is still too small and less dynamic to overcome CMV: paradoxically, high monocytosis (i.e., pool of target cells) promotes faster replication (Figure 4), and probably in this manner corticoids (Figure 5). Thus, the best treatment for CMV (in fact restoration of virome homeostasis) is to reduce immunosuppression, because ganciclovir therapy may be ineffective as described by Zhu et al. [30]. A good example of this is the much lower incidence of CMV disease in patients undergoing AIDS treatment, i.e., with immune reconstitution.

No in vitro method shows such elements of the infectious process as the incubation period, portal of entry, or interaction with myelopoiesis. Furthermore, pharmacokinetics, elimination of dexamethasone (potent and short-acting corticosteroid), and its pleiotropic effect on monocytes do not have a simple experimental counterpart [43]. Animal models, on the other hand, are not complex systems such as HSCT or primary immunodeficiency. Patients infected with CMV usually have multiple co-morbidities with various therapeutic regimens, usually with steroids. Primary immune response may be observed during secondary infection (and vice versa) and human CMV exhibits vigorous species specificity [3,14]. Translational medicine and a stricter definition of basic term such as monocyte/monocytosis [24,25,26,32] (Figure 2 and Figure 3) and virus replication (as an exponential function (1), Figure 3 and Figure 4) may allow a deeper description of CMV virome and cytomegalovirus disease as an example of dysbiosis [1].

Our study has many limitations, such as too few patients and a simple technique. A very restrictive criterion in retrospective analysis such as the exponential increase in viral load is one of the basic ones, although it brings us closer to in vitro research, e.g., study by Taylor-Wiedeman J. and coworkers with induction of endogenous human cytomegalovirus gene expression after differentiation of monocytes under influence comparable steroids dose [16]. Secondly, we have not investigated the simultaneous effects of both CMV and steroids on monocytosis (synergistic or not). It is difficult to plan a clinical trial in which long-term steroids (equivalent to 20 mg of dexamethasone) are directly tested during CMV infection (so effects of steroids and CMV were observed separately). On the other hand, their influence not only on lymphocytes but also on monocytes with increase of pool of target cells seems to be noticeable (Figure 2 and Figure 5). The monocytosis is probably a missing link between glucocorticoids and CMV reactivation (virus replication). Furthermore, the growing number of experimental studies does not coincide with the progress in diagnostics, symptomatology, and terminology (i.e., definition of crucial stages and microbiome) and more homogeneous sample of subjects [1,2]. The development of CMV disease can be divided into several stages. A better laboratory definition of CMV replication and disease development, such as that proposed here, as the exponential growth of viremia, could facilitate further progress in the clinical situations. The latest epidemic shows that basic definitions, such as colonization, infection, symptomatic infection, and infectious disease, and therefore the nomenclature and differentiation of infectious diseases should be clarified and more strictly applied based on the clinical and laboratory criteria, i.e., the host [1].

## 4. Material and Methods

### 4.1. Material

The active CMV disease records were reviewed and analyzed retrospectively from 942 patients’ history [47]. Cases were identified either at the time of hospital admission or while analyzing the medical record at follow-up appointment at the outpatient clinic for diagnosis of immunodeficiency (primary or secondary), as a control post HSCT or residual disease monitoring after therapeutic interventions in leukemia/lymphoma. The material for this study was derived from a group of 160 CMV-positive patients with positive CMV viremia whose peripheral blood samples had been analyzed. Sequential analysis of WBC, peripheral blood monocytes, C-reactive protein, and infectious status (HSV, VZV, EBV, and CMV viremia, bacterial or fungal culture) were performed. It is recommended that studies report separately cases where CMV disease is found with or without co-pathogens with details given on the co-pathogens [2] (Table 2).

**Table 2:** Patients’ characteristics.

Characteristics	All Participants (*n* = 160)		
**Median age (range)**	45 (20–65)		
**Sex**; Man (%)	83 (52%)		
**Mean viral load** (copies per 100,000 nucleated cells)	950 (100–590,000)		
**Underlying disease**			
**-Primary immunodeficiency**	45		
**-Secondary immunodeficiency**			
**after HSCT**	54		
**lymphoproliferative disease (NHL, HD)**	48		
**cancer**	16		
Exclusion criteria *:			
Bacterial infection (Gram-negative)	52 (44)		
C-reactive protein without known bacterial specimen	35		
HHV6	5		
EBV	36		
HSV 1/2	8		
VZV	2		
Fungal	4		
Tuberculosis or other mycobacteria	2		
Current therapy with known myelotoxic drug **	12		
Lack of content or uncooperative patient	3		
	↓		
**After exclusion**	**Normal subjects** **(*n* = 20)**	**Included** **(*n* = 16)**	**Steroids subgroup** **(*n* = 8)**
**Median age (range)**	48 (20–72)	38 (25–60)	40 (30–55)
**Sex**; man (%)	10 (50%)	8 (50%)	3 (38%)
Mean viral load	31 (6–89)	5875.50 (100–22,293)	28 (0–30)
Median monocyte level *** (range)	400 (155–680)	1550 (600–2400)	543 (74–2808)

A small group of patients (homogeneous sample of subjects) was characterized with CMV disease with virus replication, i.e., exponential growth of viremia. For comparison, a group of normal subjects with active replication and CMV disease with anti-CMV IgG and latent form of infection was presented. Steroid subgroup included eight patients, but during a period of time when the result of viremia was negative (less than 100 copies in two or more measurements) and the patients received steroids because of various indications. * In several patients, two or more exclusion criteria were frequently observed. ** isotretinoin, ganciclovir, metronidazole, macrolide, chloramphenicol, or tetracycline. *** monocyte level in hematology analyzer examination.

Therefore, we had a small selection of patients and that is why we compared our data with simple experimental (in vitro) models where usually a single factor was analyzed. Due to the usual occurrence of coinfection most of patients were disqualified from the analysis: concomitant viral (especially by EBV and other herpesvirus), bacterial, fungal, or rickettsial infections were excluded.

The presence of CMV in the blood, together with symptoms and/or signs, is not sufficient for the definition of either proven or probable CMV disease at any other site [2]. As shown in Figure 1, about 1000 virus copies were obtained sporadically. For this reason, the cytomegalovirus disease and CMV replication were time-lapse controlled. Patients were included when exponential growth of CMV viremia was observed in 2 or more CMV-DNA analyses (the values form a geometric progression—see Section 4.2.1). Therefore, only patients with short time of significant CMV replication and unquestionably active CMV infectious process were qualified with minimal influence of other pathogens. After the significant growth of viremia, patients received standard gancyclovir or valgancyclovir therapy [15,21].

Because contraindications in long-term glucocorticoids treatment are acute viral diseases (especially major viral infections) as well as severe primary immunodeficiency, it was difficult to observe patients with exponential increases in viral load and long-term steroid use simultaneously. Thus, in our study, we took a different approach: instead of examining the effect of steroids on CMV replication, we examined their dose-dependent effect on monocytosis, and then the relationship between CMV and monocytosis (Table 2).

In further long-term observation of included patients (*n* = 16) in various periods and for various indications (GVHD, severe drug hypersensitivity reactions, allergic conditions, lymphoid interstitial pneumonia, cerebral edema), steroids were used (often in one patient several times, in different doses with at least a month break). The 8 patients from 16 included have received steroids at least two weeks (steroids subgroup) and were compared with another 8 patients and with anti-CMV+ healthy control. Due to the inhibition of the immune response by steroids and the pleiotropic effect, the viral load was time-lapse monitored before and during glucocorticoids therapy. Treatment with steroids was not initiated when patients were herpesvirus, fungal, or other infectious diseases positive according to standard operating procedure and product characteristics. 

The effects of steroids were tested in the period of 2–3 weeks after the start of the therapeutic regimen. The studies were compared with the control group of patients without sign of immunodeficiency, not receiving ganciclovir or steroids, and having repeated CMV negative viremia tests (Table 2), and presented as “normal subjects” on Figure 2.

### 4.2. Methods

#### 4.2.1. Estimation of Burden of CMV Disease

Quantitative CMV DNA analysis in peripheral blood cells was determined using real-time PCR with Light Cycler II (Roche, Mannheim, Germany) and expressed in numbers of CMV copies in 100,000 nucleated cells of the whole blood as described previously [14,15,21]. For strict evidence of viral multiplication, virus replication in our situation was defined as exponential growth of DNA-viremia. Exponential function of viremia with various bases (b) that are a positive real number higher than 1 and whole copy CMV number as values of function.

f(x) = b^x^, when b > 1; x > 0 (1)

In our study, we used the common logarithm, i.e., with base 10 (that is b = 10) (Figure 4).

Obviously, their exponential increase makes the absolute number of CMV copies significantly dependent on the timing, blood sampling, and frequency of measurement, especially in patients after HSCT. It should be noted that latest evidence suggests that the detection of virus, antigen, or DNA in blood does not mean that CMV is replicating in blood [2].

#### 4.2.2. Monocyte Analysis

The whole blood analysis was performed to avoid possible biases through artificial stimulation of monocytes arising from preanalytical steps by mononuclear cell separation (Ficoll-density gradient centrifugation), sample cryopreservation [48,49]. To obtain higher precision and higher accuracy, no wash procedures were used [23]. Such probes preserve the “native environment” including cytokine-produced cells, hormones, but also administered drugs that were removed by washing in other models [50]. Forward and side scatter (FSC vs. SSC) were used initially to remove contamination and non-cellular elements. Preliminary study consisted of FSC/SSC analysis of monocyte population and comparison with other techniques. Further on, the leukocyte analysis was assessed in whole blood by flow cytometry using classical CD45 gating as described previously [14,21,22,24,26,28]. In brief, the panel of monoclonal antibodies (mAbs) for the detection of surface CD45, CD14, and HLA-DR was purchased from Becton-Dickinson (San Jose, CA, USA). Cells were stained for 30 min at room temperature. Human monocyte population was selected from whole blood based on gating on live CD45+ (LCA leukocyte common antigen) expression and SSC [21]. The cells were analyzed with FACScalibur Flow Cytometer (Becton-Dickinson), using CellQuest software for data acquisition and analysis. Data were expressed as cell count per 1μL. The isotype control and the specific antibody were derived from the same process.

For comparison, monocyte counts were determined from the same samples using the hematology autoanalyzer. The complete blood count (CBC) was performed with the Sysmex XN-2000 or ABC-Micros and compared to monocyte count on the basis of a primary CD45/SSC order to give numbers of gating procedure. May–Grünwald–Giemsa stain (MGG smears) was performed. Experienced hematologists carried out differential counts on MGG slides. It is currently a technique recommended by Clinical and Laboratory Standards Institute as a means to generate a reference differential count in method comparisons [26,27,28]. Pearson coefficients were used for comparison between manual counting, cell counter counts, and flow cytometry. 

## 5. Conclusions

Although the monocyte nomenclature, definition, and counting in clinical studies are still an open issue (based on morphologic/cytometric phenotype or ontogeny) [24,25,26,27,28,31,32,33], the morphology-based approach as a basis of medical interventions correlates with cytometry (Table 1). Blood smear may still be useful due to the availability and recent useful observation described by Tak and coworkers [36], and the expression of CD14 on CMV-infected monocytes (Figure 3) requires further studies. Until now, significant CMV-induced monocytosis observed here has had no counterpart in in vitro studies. Exponential growth of CMV viremia and time-lapse analysis may be a new approach ensuring greater compatibility with experimental (in vitro) studies and more adequate terms for various phases of infectious process (colonization→ infection→ latency ↔ infectious disease). In contrast to single results (Figure 1) and latency (Figure 2) CMV kinetics (hence exponential relationship) create more reliable image of the new, extremely dynamic CMV↔ monocyte interaction (Figure 4). The last crucial retrospective analysis showed that CMV viral load kinetics provided good surrogate endpoints after allogeneic transplantation; thus, this approach is a newly developed method in translational medicine [51]. 

In the era of increasing use of dexamethasone (e.g., in SARS-CoV-2), the dose-dependent effect on monocyte level (Figure 5) seems to be one of the key factors in the clinical setting. The slight influence of the dose equivalent to 5 mg of dexamethasone (Figure 5) may be a precious hint, which should be converted (after large-scale studies) in immune-transplantology. Further studies will allow to show whether the high monocytopoiesis or reduction in expression of adhesion molecules is the main cause of monocytosis. 

## Figures and Tables

**Table 1:** Relationship between four most popular methods of monocyte analysis and counting.

	Analyzer	CD45/SSC	CD14
Manual	0.895264	0.932088	0.92599
Analyzer		0.986452	0.953596
CD45/SSC			0.971847

## Abbreviations

CMV	cytomegalovirus
CBC	complete blood count
FSC/SSC	forward and side scatter
WBC	leukocytes = white blood cells
LCA	leukocyte common antigen
NBC	nucleated blood cells
MGG smears	May–Grünwald–Giemsa stain
M-CSF	macrophage colony-stimulating factor
PRRs	pathogen recognition receptors
WBC	white blood cells
